# Pre‐Procedural Safety Screening in Diagnostic Imaging Across England: A Freedom of Information Study

**DOI:** 10.1111/jep.70616

**Published:** 2026-09-25

**Authors:** Martine Ann Mallinson, Bryony Brown, Beverly Snaith

**Affiliations:** ^1^ University of Bradford Richmond Road Bradford UK; ^2^ Department of Radiology, Mid Yorkshire Teaching NHS Trust Pinderfields Hospital Wakefield UK

**Keywords:** contraindications, diagnostic imaging, document analysis, ionising radiation, Magnetic Resonance Imaging, patient safety, questionnaires

## Abstract

**Rationale:**

Patient safety screening forms are a way of identifying medical history and contraindications prior to diagnostic imaging examinations. Current utilisation of patient safety forms and variation in their design is unclear.

**Aims and Objectives:**

Adopting a pragmatic research approach, we aimed to explore pre‐procedural patient‐completed safety screening forms from imaging providers across England to identify similarities and variation in documents currently used in clinical practice.

**Method:**

A freedom of information (FOI) request was sent to all acute National Health Service (NHS) Trusts in England and Independent Service Providers (IS) between 6th July 2025 and 22nd July 2025. Organisations were asked whether screening forms used across the diagnostic imaging were completed on paper or through digital methods. Paper versions requested for analysis. Documents have been assessed with regards to procedure or purpose and design.

**Results:**

FOI responses were received from 126 NHS organisations and three IS providers, an overall response rate of 83.2%, with 91.3% of the NHS organisations responding. The average response time was 21.7 working days (range 2–106), with 72 organisations (55.8%) responding within the anticipated 20 working days. Organisations most commonly use paper forms, with only one organisation confirming that all pre‐procedural checks were completed using digital forms.

**Conclusion:**

This study has drawn on data obtained through the FOI Act. Current patient safety screening forms are largely paper‐based and demonstrate inconsistencies which may influence their interpretation and lead to errors. Future studies should evaluate their use in practice to inform evidence‐based developments in this area.

## 1 Introduction

Across the healthcare sector patient safety is paramount, this includes diagnostic imaging where practice is designed to minimise harm [1, 2]. With advances in technology and interventions, safety processes are essential and the imaging workforce is central to this increasingly complex speciality [3, 4]. Given the hazards and clinical risks associated with different imaging modalities, safety standards and pre‐procedural patient screening is commonplace internationally [1, 2]. Screening of patients, and accompanying carers is usually to confirm the medical history, demographic information and establish potential contraindications to the examination. Safety practices are commonly focussed around the risks of ionising radiation, particularly to the foetus, to identify implants and ferrous foreign bodies prior to Magnetic Resonance Imaging (MRI) and risk factors for adverse reactions or renal insufficiency prior to contrast enhanced imaging [2]. Although the processes that occur within individual hospital settings is not known, anecdotal evidence suggests that many organisations expect the patient to complete a paper form which is subsequently scanned onto the electronic imaging record. With 49.9 million imaging tests performed across England in the year to March 2025, approximately three quarters of these are expected to have involved pre‐procedural screening documents [5]. This would therefore represent significant activity for the system and workforce. Germane to this the UK National Health Service (NHS) has prioritised analogue to digital as one of the three major ‘shifts’ in its 10‐year health plan [6].

International variation in safety processes and screening forms have been reported in the literature [7]. The processes differ in the timing of screening based on who is involved in the process (patient only vs. patient with staff) or multi‐step screening processes taking place in advance and/or on the day of the procedure [7, 8, 9, 10, 11, 12]. Documentation of patient safety checks can vary across and within organisations and be dependent on patient type with paper versions the most common format [7]. The process of collating patient medical history to maintain safety during diagnostic imaging examinations found to result in delays [11, 13], particularly relating to missing or inaccurate information [14, 15, 16]. We know from previous surveys that practices, and indeed even the questions used, vary [17, 18, 19], however, the scale is not known currently. Understanding real world practice is the first step in gaining consensus on the approach and enabling the development of digital processes. The aim of this study was to identify commonalities and differences in pre‐procedural screening documentation used in English diagnostic imaging services.

## 2 Methods

### 2.1 Data Collection

The study adopted a pragmatic research philosophy focussed on addressing the real‐world research problem through the most appropriate method [20]. Recognising that knowledge is shaped by social and cultural and historical contexts, this approach has been used to reflect on what can be learned from current practice to inform the development of future solutions [21]. The pragmatic approach which is problem driven, not method‐driven underpinned the preference to employ a freedom of information approach to maximise imaging service provider engagement. Institutional ethical approval was received prior to data collection (EC28695).

Given the challenges in cross‐sectional survey responses and the complexity in imaging service configuration, a Freedom of Information (FOI) process has been employed. Often adopted by journalists, this method is historically poorly utilised for academic research [22]. The process is available in many countries but will vary according to jurisdiction and legislation but enables access to data held by public bodies not published elsewhere [22]. Similar approaches have been utilised in health research including exploring service provision [23, 24, 25], technology adoption [26] and patient outcome measures [27]. In our study the FOI request sought to identify the prevalence of analogue and digital methods of screening across England and to understand the scale of variation in forms used in practice. All NHS Trusts providing imaging services (*n* = 138) and independent sector (IS) providers (*n* = 17) of diagnostic imaging to the NHS were identified through website searching. The FOI request was submitted to NHS providers by email or through a defined portal signposted on organisation webpages between 6th and 9th July 2025, with IS requests completed on 22nd July 2025. In line with the FOI act [28] requirements the request was sent by the lead author asking (1) whether pre‐procedural screening forms for imaging utilised paper or digital media (tablet or web‐based methods), and (2) for copies of any patient‐facing paper forms to be returned by email. As the information was being requested for research purposes, reminders were sent to non‐responders after 27 working days, with a second reminder was sent at 44 working days. Some organisations requested an extension the expected 20 working day (excluding weekends and bank holidays) response [28].

Organisations were excluded from if they did not provide imaging services, e.g. mental health or community NHS Trusts. Additionally, referral forms and pre‐procedural checklists forming part of a staff‐facing safety briefing, such as in interventional radiology [29, 30], were also excluded. Other staff completed forms where safety or medical history questions were asked of the patient as part of the examination, with or without their signature, were included.

Free text FOI email responses were transcribed verbatim into Microsoft Excel (Redmond, Washington, US). Where multiple responses were received from the same organisation any attached documents were reviewed and duplicates removed. Each imaging provider was charted on a separate line and provided a unique study identifier. The returned document titles were transcribed under a priori categories related to the primary purpose derived from researcher consensus and the available literature, for example modality safety, drug administration, pregnancy status. Where the documents did not have a name included, these were all entered as ‘untitled’.

### 2.2 Data Analysis

Tabulated data was analysed for document format, number of forms returned (within and across organisations), document titles and use of key terminology. The free text responses were analysed thematically using Braun & Clarke's systematic process [31]. Interpretation involved familiarisation with the data set through reading and transcribing and coding of FOI responses before the generation, checking and refining of themes. The development of analytical constructs was performed by an individual researcher with independent review by a second researcher. Representative anonymous quotes have been included using the study identifier (e.g. O12). The study was performed and reported in accordance with the strengthening and reporting of observational studies in epidemiology (STROBE) checklist [32].

## 3 Results

FOI responses were received from 126 NHS organisations and 3 IS providers, an overall response rate of 83.2%, with 91.3% of the NHS organisations responding. The average response time was 21.7 working days (range 2–106), with 72 organisations (55.8%) responding within the FOI expected timeframe. Overwhelmingly, organisations use paper forms for pre‐procedural checks, with only one NHS provider confirming that all checks were completed using digital forms. Many stated that paper forms are stored electronically, although the location varied and included the radiology information system (RIS), the picture archive and communication system (PACS) or wider electronic patient record (EPR).

Safety screening forms or checklists which are completed by patients prior to a diagnostic imaging examination are in paper form and are scanned into our Picture Archiving and Communication Systems (PACS) against the patient attendance.

(O8)

Some processes appear convoluted.

Most forms are printed from the Radiology Information System (Soliton) and completed on paper by/with the patient before being scanned back into the patient's record.

(O155)

Inconsistent processes were noted across the whole imaging pathway with some stating that the referral forms are also printed.

Patient signs the referral form to confirm they are not pregnant – This is then a scanned document on the patient's file.

(O114)

In addition to the one digitally enabled organisation, another 16 organisations confirmed they have some digital processes in place, although many stated that this was not consistent across modalities or examinations.

Contrast safety questions for both CT and MRI are on our CRIS tablets as well as paper but for particular examinations that require more information, we have additional paper questionnaires that we have been unable to add to the tablets.

(O13)

MRI uses a tablet and is digital format (Mobile CRIS) for all patients with the exception of IP [inpatient] referrals where there is cognitive impairment when a paper form is completed.

(O68)

patients’ complete safety screening forms and checklists, some of which are in paper format, and some are in digital format.

(O65)

Two additional respondents described they have plans to go digital whilst another reported that they are currently exploring this within a single modality at one of the hospital sites.

The Trust are currently piloting a digital copy if the CT checklist at [hospital] site, with patients completing the questions through iPads.

(O55)

Regardless of format the pre‐procedure processes also vary, with some screening forms sent out by post in advance of appointments.

these are sent to the patient by email/post. We then expect the patient to bring these in with them for their appointment.

(O97)

The MRI general safety screening form is typically sent to patients in advance for completion. Patients are advised to contact the department by phone if they have any safety concerns. (O30)

Whereas in other organisations the patient completes the form on arrival.

all paper safety screening forms/checklists which are completed by patients prior to a diagnostic imaging examination within Radiology.

(O142)

Whilst for some the staff ‘interview’ the patient using defined questions

Imaging [staff] ask the relevant questions and patients are then asked to sign these.

(O145)

Forms are completed by Radiographer during verbal safety checks and signed by patient.

(O140)

every consent form is checked through and signed on the day with an MRI safety trained radiographer/nurse, using a tablet.

(O43)

Of the 155 eligible organisations contacted, 127 organisations returned at least one of their paper‐based forms and a further 2 IS providers' forms were returned by NHS Trusts with whom they contract services. A total of 824 documents were supplied by the 130 organisations and these ranged from 1 (*n* = 7) to 25 forms (*n* = 1). However, the documents reviewed are acknowledged not to represent all the different documents in clinical use, with one site stating that *there are dozens of variations and so it is not possible to send a comprehensive example* (O50).

All supplied documents were in the English language only. The titles varied significantly, including a substantial number (*n* = 51) which had no document title. ‘Safety’ was included in the title on 196 documents, with another 57 using the term ‘screening’. The procedure and/or purpose was often stated, with the most used terms being ‘questionnaire’ or ‘checklist’ (Table 1) although sometimes multiple terms were included in the same title, for example ‘Magnetic Resonance Imaging Department Patient History and Safety Screening Form’. Some documents were titled ‘information’ but required the patient to answer a series of questions related to their medical history.

**Table 1:** Summary of terms included in the titles of the returned forms.

Term used (suffixes)	Occurrences
Agreement	6
Assessment	7
Authorisation	4
Check (form/list/sheet)	157
Consent (form)	78
Declaration	14
Disclaimer	7
Enquiry (form/questionnaire)	3
Pathway	4
Proforma	5
Procedure (list/record)	29
Questionnaire	281
Record (sheet)	30
Status (check)	38
Waiver (form)	2
Work (flow/sheet)	7

*Note:* The bracketed terms demonstrate how the term is used as a stem (e.g. worksheet).

The largest number of paper forms were related to computed tomography (CT) (*n* = 253) and MRI (*n* = 194), although forms were supplied across a number of different imaging modalities (Table 2). The safety screening processes were most commonly linked to the clinical risk of the environment or administration of drugs, including contrast media. Many sought details of previous surgery, risk factors such as reduced renal function, or relevant medical history to guide the procedure or interpretation of the resultant images. Two forms sought detail on specific risk factors for lung cancer including smoking status and symptoms, one for CT and another X‐ray.

**Table 2:** Pre‐procedural screening documents used across organisations by modality.

Modality procedure/purpose	Organisations supplying forms *n* (%)	Number of forms returned	Number of different titles
Bone densitometry (DXA)	**70 (54.2)**	**76** *	**52**
Medical history	66 (51.1)	69*	47
Safety	9 (6.9)	9	8
Computed Tomography (CT)	**108 (82.4)**	**253** *	**189**
Safety (inc contrast)	97 (74.0)	114*	83
CT cardiac	50 (38.2)	54*	41
CT Colonography	68 (51.9)	76*	59
Other	8 (6.1)	9*	9
Fluoroscopy	**14 (10.7)**	**19** *	**19**
Hysterosalpingogram	6 (4.6)	6	6
Proctogram	3 (2.3)	**3**	**3**
Other	7 (5.3)	10*	10
Magnetic Resonance Imaging (MRI)	**113 (86.3)**	**194**	**136**
Safety	110 (84.0)	140*	85
Cardiac	9 (6.9)	**9**	**9**
Contrast	21 (16.0)	22*	21
Pregnancy	10 (7.6)	11*	11
Other	11 (8.4)	12	12
Mammography	**9 (6.9)**	12	12
Implants	4 (3.1)	4	4
Contrast	6 (4.6)	6	6
Other	2 (1.5)	2	2
Nuclear medicine	**30 (22.9)**	**56** *	**54**
General	13 (9.9)	13	12
Bone	9 (6.9)	9	8
Cardiac	8 (6.1)	9*	9
Sentinel Node	3 (2.3)	7*	7
Therapy	3 (2.3)	4*	4
Other	7 (5.3)	14*	14
Ultrasound	**3 (2.3)**	3	3
X‐ray	**3 (2.3)**	3	3

*Note:* *Includes multiple forms from same organisation. Bold values are modality sub‐totals.

Eighteen of the forms related specifically to paediatric patients returned by NHS Trusts with, and without, a dedicated children's hospital. Half of the forms focussed on MRI safety screening and a further five were dedicated to CT safety. The forms often mentioned paediatric, child or children in the title but appeared to vary in the intended audience with some specifying guardian completion. Others included child friendly language and images with the appearance suggesting that they have been developed for completion with the patient where possible.

The pregnancy status of the patient was a common pre‐procedural process with 128 forms unique to this question. In addition, a question was also included on many modality‐specific questionnaires. Patient confirmation of pregnancy, or possible pregnancy, but agreeing to go ahead with the imaging procedure was the focus of 12 forms. Twenty‐two different organisations supplied triage type forms which just required a yes/no answer regarding the possibility of pregnancy and often the patient signature. Another 27 organisations expected the patient to detail the date of the last menstrual period (LMP) and confirm that they were not pregnant, with additional forms designed for high radiation dose examinations (*n* = 14). In comparison, an inclusive pregnancy status (IPS) questionnaire designed to establish sex at birth and the potential for pregnancy regardless of gender was supplied by 45 organisations with variations for high dose procedures (*n* = 3). Finally, six forms were provided specific to nuclear medicine due to the need to also identify whether the patient was chest or breast feeding due to the administration of radiopharmaceuticals.

Drug‐related forms were supplied by 34 organisations, including 56 different forms for use of contrast media, analgesia or antispasmodics. Commonly, these included relevant contraindications, risk factors and an administration record. There was inconsistency as to whether the patient and/or the radiographer was required to countersign the form. This subject was also included as a defined section on some modality‐specific documents.

Although the majority of forms related to patient screening, those acting as an accompanying carer and comforter were also subject to pre‐procedural safety processes with 21 organisations supplying forms tailored to this group. Eleven of these covered safety checks for those entering the MRI environment, with a further 10 forms related to ionising radiation examinations.

## 4 Discussion

In seeking to explore pre‐imaging screening practices across England the researchers adopted the FOI request as research method. There are documented challenges with accessing information held by public bodies for the purposes of research, including unavailability of data, application of exemptions of the act and adherence to timelines [33, 34, 35, 36]. However, as seen in this work this method can elicit a high response rate in comparison to a cross‐sectional survey [37], and enables robust comparison of standardised data [38]. Although commercial companies are not bound to respond but where they supply services on behalf of a public authority and hold information in connection with those services, information may be requested under the FOI act [39]. The public authority is then responsible for responding to the request on the commercial companies’ behalf. The additional challenge of seeking IS data is the dispersed nature of services, with single organisations having facilities across multiple geographic regions and/or in collaboration with NHS providers.

At an organisational level, the data presented represents just a snapshot of documents. In some cases, only a selection of the available versions was supplied as a representation of the variation across different areas of practice. However, the study has allowed insight into the range of pre‐procedural forms used across the country. An advantage of this has been the ability to assess inconsistencies in terminology used in pre‐procedural screening within and across organisations with multiple names for the forms which can only add to the confusion. Although further content analysis of forms collated is planned based on imaging modality, it is safe to say that the differences in document titles imply inconsistency in focus and therefore content. Safety screening forms are often guided by published safety guidance [19] however there is no currently consensus on suitable risk factor tools and question sets for modality‐specific practices [40]. This variation has implications for the pre‐procedural screening process which essentially forms part of the informed consent and shared decision‐making process [14]. Consent or permission for a medical procedure must be informed and given voluntarily, meaning that pre‐procedural documentation should be easily understood by patients. The use of jargon and non‐standard NHS terms such as ‘waiver’ and ‘disclaimer’ identified in this study adds to the complexity because their definition and inference are not explicit or understood by the patient completing the form or anecdotally by staff taking responsibility by co‐signing such documents prior to the examination. Evidence shows that a lack of standardisation and structure in the pre‐procedural screening process can result in near miss events and safety incidents [8, 13]. Building on this previous work in MRI safe practice, this imaging‐wide study validates recent reporting of the exacerbating factors specific to this context. This includes the fast‐paced and challenging clinical environment which places pressure on the imaging workforce to carry out pre exposure checks, the use of multiple procedures and processes across different sites within the same organisations, and the use of mobile facilities and visiting staff in some specialised modalities which provide opportunity for mistakes [41]. Common high‐risk failures include missed pregnancy checks and failure to identify carers and comforters [41]. As confirmed by this study, carer and comforter safety forms for ionising radiation examinations were returned by a only small number of organisations and may not be widely adopted. Further, some evidence of specific safety screening practices for paediatrics and other vulnerable groups, such as those lacking capacity, were identified in this study although the format of these documents and responsibility for completion varied.

In concordance with previous international studies, this study demonstrated variation with regard to when the screening form is completed, with options of the screening form being sent in the post with the appointment letter for completion before the appointment, completed by the patient on arrival in the department, or with a staff member on the day of the imaging test [7, 8, 9, 10, 11, 12]. These differences in pathway (including multi‐step processes where the responses are confirmed at several stages to provide a fail‐safe) are likely to result in different costs per episode for delivery. The NHS aspires to be paperless with its digitisation programme introduced in 2021 [42]. Despite diagnostic imaging being a technology‐enabled environment in relation to image acquisition, reporting, storage and distribution, the findings of this study corroborate that safety screening processes are still predominantly paper based. To date there has been limited digital engagement in this context, with only a single organisation having made the shift to entirely digital methods for pre‐procedural safety screening although a small number of organisations confirming aspirations. A recent US single‐centre study reported on the positive impact of an online MRI screening platform and found a reduction in the number of post‐arrival cancellations [43]. Previous studies examining pre‐surgical patient consent processes have demonstrated that a digital pathway can be cheaper than paper [14]. A full costing analysis comparing paper screening forms to a digitally enabled process has not been undertaken at this time, however our findings suggest that at least 90% of organisations are currently using paper forms. When the number of examinations requiring screening is considered these amounts to more than 38 million sheets, or 76,000 reams, of paper. To note, this is printing one sheet of paper only, where some of the forms submitted were multiple pages long and so is likely to be an underestimation. We do not fully know what cost burden this activity has on imaging staff time including support in patient completion and management of outcomes and a full cost utility analysis of pathway would be required. Digitisation across all aspects of imaging and diagnostics has been recommended to drive efficiency, standardise data and deliver seamless care across organisational boundaries [44]. Advantages also include improving sustainability, expanding patient access, and capturing health and care information digitally at the point of care to support clinical‐decision making and reduce health risks [6]. The transition away from paper would need to maintain and assess digital equity [45], however this would support patient‐centred care and reduce disparities between patient groups supporting communication and overcoming language barriers highlighted as important for consent and safety during imaging [46]. Although not asked directly as part of the FOI request, no responding organisations reported having multilingual options available for their imaging pre‐procedural screening forms. This practice mirrors the evidence base with only one previous study reporting the development and implementation of a digital multi‐language questionnaire within the imaging context [47].

## 5 Limitations

This study has a number of limitations. We acknowledge the number of organisations responding beyond the FOI expected 20 days but recognise that the request was distributed at the start of the UK summer holidays. Some organisations requested a formal extension thus justifying the continuation of data collection. On a form basis, there may be some geographical spread, but the location has not been reported due to the 90% response rate. Only data provided by organisations has been analysed and therefore it is recognised this does not present a complete picture of pre‐procedural screening and further research is required to understand the processes in clinical practice. It is also acknowledged that some organisations place the initial responsibility for safety screening on the referrer with the potential for outcomes being confirmed at the examination [7, 8, 48], these were however considered beyond the scope of the study. However, the data collected has been sufficient to confirm significant variation across services. The manuscript summarises the number of unique titles, but the content and design of the forms have not been part of this analysis and will form part of future work.

## 6 Conclusion

Our findings highlight that current UK pre‐procedural imaging screening processes are currently paper based despite national priorities for digital transformation and aims to improve sustainability in healthcare. Organisations are reliant upon manual processes for capturing and documenting elements of patient safety and opportunities to reduce costs, staff burden and improve workflows require further consideration. Current screening forms demonstrate inconsistencies in administration, essence and clarity for those completing them which may influence their interpretation and lead to errors, with the imaging environment also contributing to clinical risk. Consensus on screening processes and specific questions relevant to the imaging modality is required, although it is acknowledged this may have to be completed on a national, rather than international, level to meet professional and regulatory guidance. Additionally, there is significant opportunity for the adoption of digital solutions, but this will require alignment with referral processes and imaging systems. Although this study demonstrates the breadth of pre‐procedural patient safety screening in this context, future studies should evaluate its use in practice to understand screening pathways, underlying mechanisms for variation and the wider consequences of adopting digital technology.

## Funding

The authors have nothing to report.

## Conflicts of Interest

The authors declare no conflicts of interest.

## Data Availability

The authors have nothing to report.
